# Magnetic Mn_5_Ge_3_ nanocrystals embedded in crystalline Ge: a magnet/semiconductor hybrid synthesized by ion implantation

**DOI:** 10.1186/1556-276X-7-528

**Published:** 2012-09-25

**Authors:** Shengqiang Zhou, Wenxu Zhang, Artem Shalimov, Yutian Wang, Zhisuo Huang, Danilo Buerger, Arndt Mücklich, Wanli Zhang, Heidemarie Schmidt, Manfred Helm

**Affiliations:** 1 Institute of Ion Beam Physics and Materials Research, Helmholtz-Zentrum Dresden-Rossendorf, PO Box 510119, Dresden, 01314, Germany; 2 State Key Laboratory of Electronic Thin Films and Integrated Devices, University of Electronic Science and Technology of China, Chengdu, 610054, China; 3 Technische Universität Dresden, Dresden, 01062, Germany; 4 Fakultät Elektrotechnik und Informationstechnik, Materialsysteme der Nanoelektronik, Technische Universität Chemnitz, Chemnitz, 09107, Germany

**Keywords:** Mn_5_Ge_3_, Ion implantation, Magnetic nanocrystals, Magnetoresistance

## Abstract

The integration of ferromagnetic Mn_5_Ge_3_ with the Ge matrix is promising for spin injection in a silicon-compatible geometry. In this paper, we report the preparation of magnetic Mn_5_Ge_3_ nanocrystals embedded inside the Ge matrix by Mn ion implantation at elevated temperature. By X-ray diffraction and transmission electron microscopy, we observe crystalline Mn_5_Ge_3_ with variable size depending on the Mn ion fluence. The electronic structure of Mn in Mn_5_Ge_3_ nanocrystals is a 3*d*^6^ configuration, which is the same as that in bulk Mn_5_Ge_3_. A large positive magnetoresistance has been observed at low temperatures. It can be explained by the conductivity inhomogeneity in the magnetic/semiconductor hybrid system.

## Background

Due to its compatibility to Si technology, Ge has attracted special attention as a host semiconductor for diluted magnetic impurity atoms. However, due to the low solid solubility of transition metals in Ge, intermetallic compounds (mainly Mn_5_Ge_3_) tend to form in the Ge host [1-6]. Mn_5_Ge_3_ is a half metallic ferromagnet with a large spin polarization [7]. By first principle calculation, large spin injection efficiency is expected by the integration of Mn_5_Ge_3_ within the Ge matrix [7]. Electrical spin injection and detection in Ge have been experimentally demonstrated [8,9]. Therefore, considerable work has been done to fabricate epitaxial Mn_5_Ge_3_ films as well as nanostructures [10-12]. The Curie temperature (*T*_C_) of Mn_5_Ge_3_ is 296 K, which can be effectively increased by carbon doping. Spiesser et al. reported the epitaxial growth of Mn_5_Ge_3_C_*x*_ films on Ge(111) [13]. When *x* is around 0.6, *T*_C_ can be as high as 430 K. On the other hand, some unknown nanoscale Mn-rich phases also form under particular conditions during molecular beam epitaxy (MBE) growth [14-19]. Those nanostructures can have a *T*_C_ much higher than 300 K. Besides MBE, ion implantation has been used to prepare ferromagnetic semiconductors as well as hybrids of ferromagnets embedded in semiconductors [20-24]. The advantages of ion implantation include compatibility with conventional Si-chip technology and lateral patterning. Patterning by ion implantation allows the synthesis of magnetic structures comprising different magnetic phases. By carbon implantation into Mn_5_Ge_3_ and Mn_5_Si_3_, Sürgers et al. obtained lateral magnetic hybrid structures in the micrometer and submicrometer range [25]. In this contribution, we report the preparation of magnetic Mn_5_Ge_3_ nanocrystals embedded inside the Ge matrix by Mn ion implantation at an elevated temperature. We identify the formation of nanocrystalline Mn_5_Ge_3_ by X-ray diffraction (XRD) and transmission electron microscopy (TEM). The magnetic, electronic, and magnetotransport properties will be reported for this magnetic/semiconductor hybrid system.

## Methods

Nearly intrinsic Ge(001) wafers (n-type with the electron concentration of 10^13^ to 10^14^ cm^−3^) were implanted with 100-keV Mn ions at 673 K to avoid amorphization. It is worthy to note that we also used p-type Ge(001) as the substrates and got similar structural and magnetic properties. We varied the ion fluence to get samples with a large range of Mn concentrations, resulting in different structural and magnetic properties. The corresponding preparation and characterization parameters are listed in Table 1. Structural analysis was performed by synchrotron radiation XRD (SR-XRD) at the Rossendorf beamline (BM20) at the ESRF with an X-ray wavelength of 0.154 nm. Magnetic properties were analyzed using a superconducting quantum interference device magnetometer (Quantum Design Inc., San Diego, CA, USA) with the field along the sample surface. X-ray absorption spectroscopy (XAS) measurements were performed at the beamline UE46/PGM-1 at BESSY II (Helmholtz-Zentrum, Berlin, Germany). Magnetotransport properties were measured using the van der Pauw geometry with a magnetic field applied perpendicular to the film plane. Fields up to 9 T were applied over a wide temperature range from 5 to 300 K.

**Table 1:** Sample identification, structural, and magnetic parameters

**Sample identifier**	**Mn fluence**	**Concentration**	**Mn**_**5**_**Ge**_**3**_**(XRD)**	***T***_**max**_**(ZFC/FC)**	**Average diameter**
1E15	1 × 10^15^ cm^−2^	0.2%	-	-	-
1E16	1 × 10^16^ cm^−2^	2%	Yes	185 K	5 nm
5E16	5 × 10^16^ cm^−2^	10%	Yes	270 K	11 nm

## Results and discussion

### Mn_5_Ge_3_ nanocrystal formation

**Figure 1:**
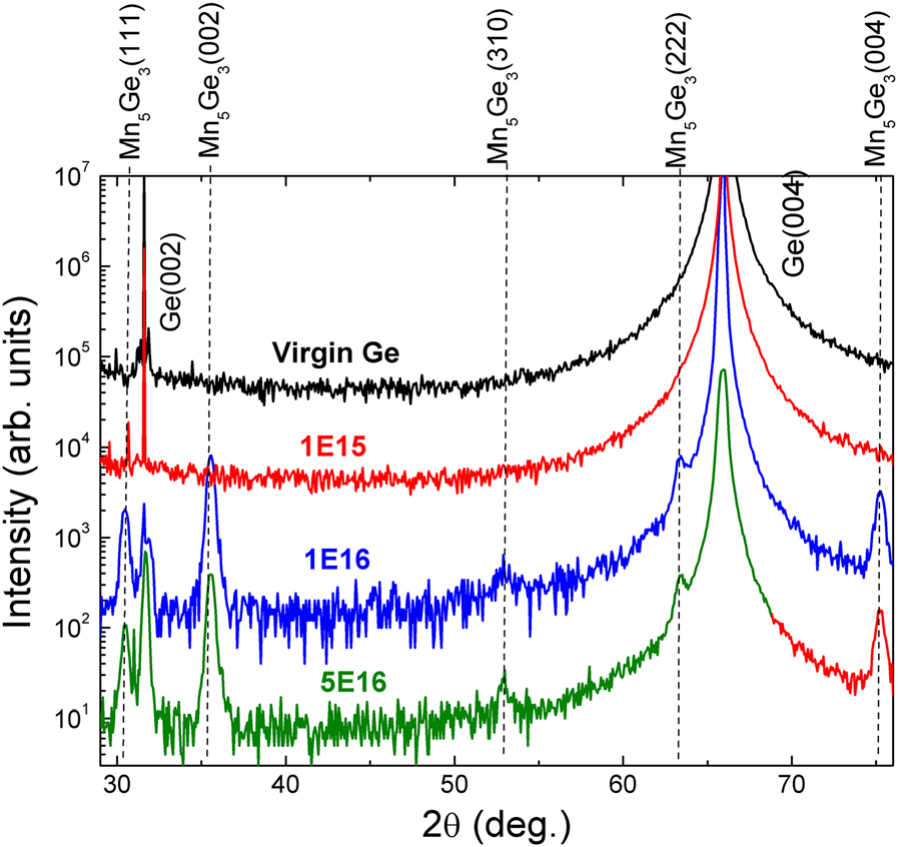
**XRD 2θ-θ scans revealing the formation of Mn**_**5**_**Ge**_**3**_**nanomagnets.** Beside the main peaks from Ge(004), the diffraction peaks of Mn_5_Ge_3_(111), (002), (310), (222), and (004) are clearly visible.

The SR-XRD 2θ-θ scans confirm the formation of Mn_5_Ge_3_ nanomagnets. As shown in Figure 1, beside the main peaks from Ge(004), the diffraction peaks of Mn_5_Ge_3_(111), (002), (310), (222), and (004) are clearly visible. Note that, compared to the work by Ottaviano et al. [20], the SR-XRD reveals more Mn_5_Ge_3_ peaks even for a much smaller Mn ion fluence due to the large flux of X-rays from the synchrotron source, which allows for the detection of small Mn_5_Ge_3_ nanocrystals. Therefore, we have to revisit the work by Ottaviano et al*.* They concluded that Mn_5_Ge_3_ nanocrystals formed by ion implantation are preferentially (002)-oriented in the Ge(001) matrix [20]. However, Zeng et al. [10] prepared Mn_5_Ge_3_ layers by molecular beam epitaxy, and they found the crystalline orientation as Mn_5_Ge_3_(001) ∥ Ge(111). The SR-XRD observations therefore led us to conclude that Mn_5_Ge_3_ nanocrystals formed by Mn implantation are indeed randomly oriented inside the Ge(001) matrix, which is also supported by the magnetic properties shown later. We found nearly isotropic hysteresis loops with magnetic field along different directions. Note that in the work of Jain et al. [26], the Mn_5_Ge_3_ nanocrystals were grown by annealing GeMn films on Ge(001) substrates prepared by MBE. Most of the Mn_5_Ge_3_ clusters (97%) have their *c*-axis perpendicular to the film plane. The accumulated literature data suggest that the growth of Mn_5_Ge_3_ nanocrystals from the Ge matrix is different from the Mn_5_Ge_3_ thin films.

**Figure 2:**
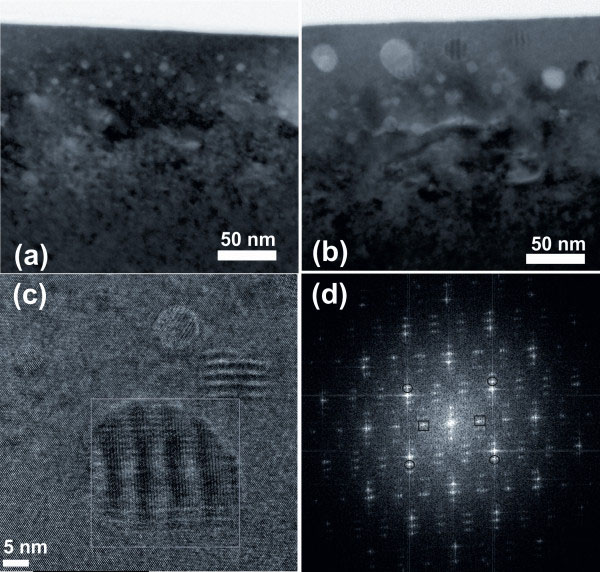
**TEM and high-resolution TEM images of samples.** TEM image of the cross section showing the formation of precipitates (**a**) 1E16 and (**b**) 5E16, and high-resolution TEM for an individual Mn_5_Ge_3_ particle (**c**) in sample 5E16. (**d**) FFT of the precipitate indicated in (c).

Figure 2 shows cross-section TEM images of samples 1E16 and 5E16. The white contrast spots are from precipitates that are located in the depth between 20 and 120 nm, which is in agreement with the depth profile of Mn ion implantation. The average crystallite size is increased from 5 to 11 nm with increasing Mn fluences from 1 × 10^16^ to 5 × 10^16^ cm^−2^. For detailed analysis, we focus on the sample 5E16. As shown in Figure 2b, the well-defined Moiré patterns are a strong indication for monocrystalline precipitates embedded in a crystalline matrix. Using high-resolution TEM, the precipitates can be identified to be Mn_5_Ge_3_, as shown in Figure 2c,d. Figure 2d is the fast Fourier transform (FFT) of the image shown in Figure 2c. The FFT reveals lattice spacings amounting to 0.298 nm (indicated by the open circles) and 0.623 nm (indicated by the open squares), which correspond to Mn_5_Ge_3_(111) and (001), respectively.

### Magnetic properties

**Figure 3:**
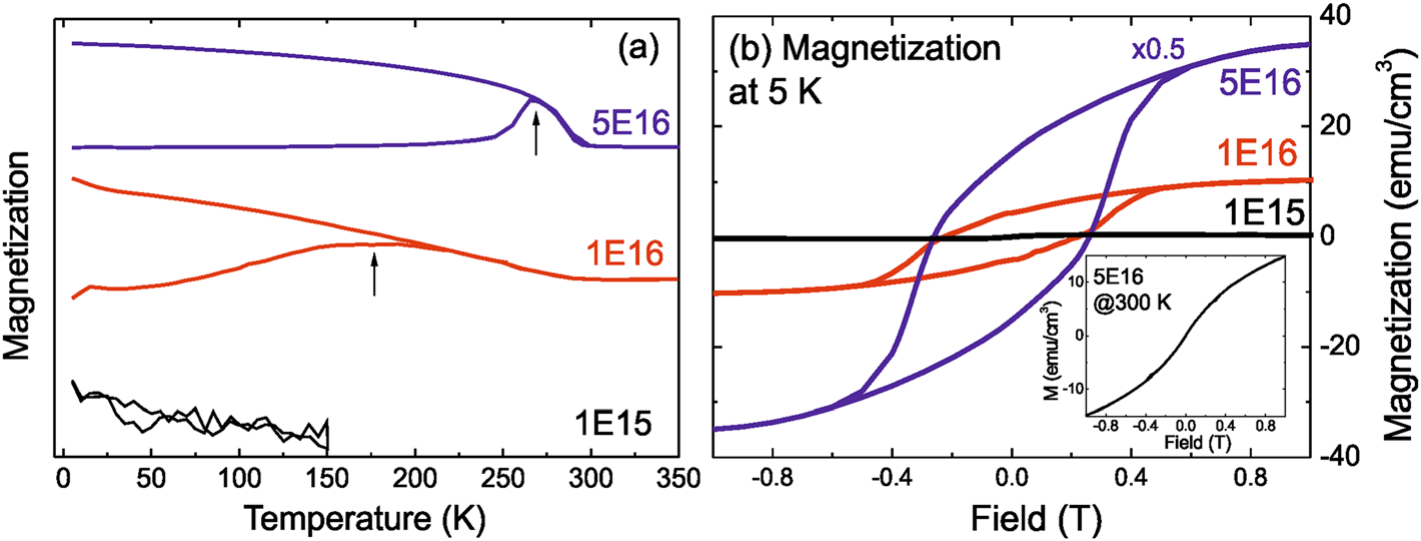
**ZFC/FC magnetization and hysteresis loops.** (**a**) Magnetization curves with an applied field of 50 Oe after ZFC/FC for the Mn-implanted Ge. The lower branches are ZFC curves, while the upper branches are FC curves. With increasing fluence, the Mn_5_Ge_3_ nanocrystals are growing in size, resulting in a higher blocking temperature. The curves are vertically shifted to increase the visibility. (**b**) Hysteresis loops measured at 5 K for Mn-implanted Ge with different fluence, and the inset shows the magnetization at 300 K for sample 5E16.

Figure 3a shows the zero field-cooled and field-cooled (ZFC/FC) magnetization curves in a 50-Oe field for different Mn fluences. The FC curve for sample 1E15 completely overlaps with the corresponding ZFC curve at around zero. Magnetic Mn_5_Ge_3_ nanocrystals can be excluded in this sample, which is consistent with the SR-XRD observation, except that they are very small and dilute beyond the detection limit of SR-XRD. For samples 1E16 and 5E16, a distinct difference in the ZFC/FC curves was observed. The ZFC curves show a gradual increase at low temperatures, peaking at different temperatures, while the FC curves monotonically increase with decreasing temperature. The width of the peaks in the ZFC curves is due to the size distribution of Mn_5_Ge_3_ nanocrystals, as shown in the TEM images (Figure 2). In this paper, we take the temperature (*T*_max_) at the maximum of the ZFC curve as the average blocking temperature listed in Table 1.

Figure 3b shows the magnetization versus field reversal (M-H) of all samples measured at 5 K. Hysteretic behaviors were observed for samples 1E16 and 5E16. With increasing Mn concentration, the saturation magnetization is increased from 10.1 to 69.2 emu/cm^3^ (by assuming the implanted depth of 100 nm), and the coercivity is increased from 0.22 to 0.26 T. At 300 K, sample 5E16 only shows field-induced magnetization (see the inset of Figure 3b). The saturation magnetization of the sample 5E16 (1E16) is 69.2 (10.1) emu/cm^3^, corresponding to around 1.5 (1.1) μ_B_/Mn, which is smaller than 2.6 ± 0.5 μ_B_/Mn as reported in the study of Bihler et al. [1], which means that not all of the implanted Mn ions form the ferromagnetic Mn_5_Ge_3_ phase.

We also compared the magnetization between the in-plane and out-of-plane directions at 5 K for sample 5E16 (not shown). In contrast to the studies of Bihler et al. [1] and Jain et al. [8], there is no detectable magnetic anisotropy. For the bulk Mn_5_Ge_3_, the magnetic easy axis is [001]. The absence of magnetic anisotropy in our samples is due to the random crystallographic orientation of the Mn_5_Ge_3_ nanocrystals.

As shown in Figure 3b, the hysteresis loop is not square-like. The distribution of coercivity field is due to the size distribution of the nanomagnets, as evidenced by the TEM images, and is also possibly due to the random distribution of the nanomagnet easy axis. According to the Stoner and Wohlfarth model for single-domain magnetic nanoparticles, the maximum coercive field gives the anisotropy field *μ*_0_*H*_*a*2_ = 0.26 T for sample 5E16. Using the bulk saturation magnetization (*M*_*S*_) for Mn_5_Ge_3_ (1,100 kA/m) [26], one can deduce the anisotropy constant: K_2_ = *μ*_0_*H*_*a*2_*M*_*S*_/2 ≈ 1.4 × 10^5^ J/m^3^, which is smaller than the value reported by Jain et al*.*[26]. Based on the Néel-Brown model, the volume for a nanomagnet V = 25*k*_B_*T*_max_/K_2_ (*k*_B_ as the Boltzmann constant), we calculate the average diameter of Ge_3_Mn_5_ clusters in sample 5E16 to be approximately 10.8 nm (*T*_max_ *=* 270 K). The average diameter is in good agreement with the results obtained by TEM. However, the average diameter for sample 1E16 deduced from the ZFC magnetization is as large as 9.5 nm, which is much larger than the value from the TEM observation.

**Figure 4:**
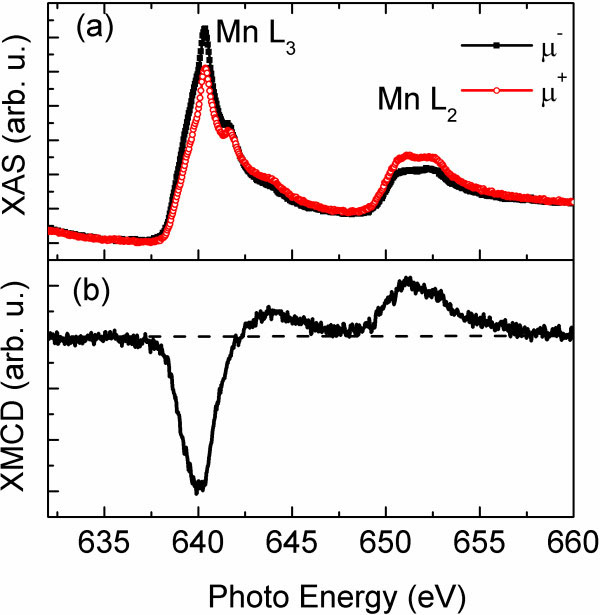
**Mn *L***_**2,3**_**X-ray absorption.** (**a**) XAS for magnetization and helicity parallel (μ+) and antiparallel (μ−) and (**b**) XMCD (μ + − μ−) measured at around 10 K under an external field of 6,000 Oe applied perpendicular to the surface.

The magnetic properties of the Mn-implanted Ge were also investigated by X-ray magnetic circular dichroism (XMCD) at Mn *L*_2,3_ edge. Right before the XAS measurements, the sample was etched in deionized water for 2 min to remove the surface oxide layer [27]. Figure 4a presents the Mn *L*_2,3_ XAS measured in total electron yield mode at around 4.5 K; *μ* + and *μ* − represent the absorption intensity with the direction of magnetization parallel and antiparallel to the photon helicity, respectively. As shown in Figure 4a, after etching, we obtained very similar spectra as was reported for ferromagnetic Mn_5_Ge_3_[28]. The XAS spectra can be classified into the 2*p*_3/2_ (approximately 641 eV) and 2*p*_1/2_ (approximately 651 eV) absorption regions. The shape of the main feature indicates the itinerant nature of ferromagnetic Mn_5_Ge_3_. On the other hand, the weak shoulders appear at 642 and 644 eV, and the doublet structure is observed in the 2*p*_1/2_ excitation region, which could be related with some diluted Mn impurities in the Ge matrix [21] or oxidized Mn [29]. Figure 4b shows the XMCD spectrum, revealing a large negative signal (approximately 641 eV) and a small positive signal (approximately 644 eV) in the 2*p*_3/2_ region and a larger positive signal (approximately 651.5 eV) in the 2*p*_1/2_ region. Note that the shoulders and the doublet in XAS spectra are hardly resolvable in the XMCD spectrum, which indicates that the oxidized Mn ions have no contribution to the ferromagnetism. According to the sum rule, the integrated intensity of the XMCD signal in the whole region is proportional to the orbital magnetic moment relative to the spin magnetic moment. In the present XMCD spectrum, the integration is nearly zero, indicating that the orbital magnetic moment is negligible for Mn_5_Ge_3_. Comparing our experimental results with the published calculations, the electronic structure of Mn ions can be assumed to be in the 3*d*^6^ configuration without spin-orbit interaction [28,30].

### Magnetotransport properties

**Figure 5:**
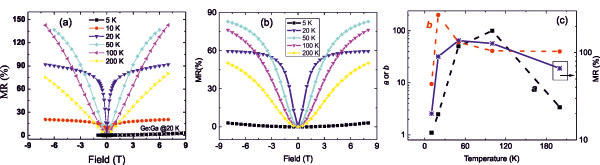
**Measured MR, calculated MR, and parameters.** (**a**) MR for sample 5E16 measured at different temperatures and the result for Ga-implanted Ge (*) is shown for comparison, (**b**) the calculated MR by considering two conductivity components, and (**c**) parameters *a* and *b* used in the FEM calculations as well as the MR data at 6 T at different temperatures.

All three samples show p-type conductivity and large magnetoresistance (MR) effect. Figure 5a shows the measurement results for sample 5E16. The MR is defined as *R*(*H*) − *R*(0)] / *R*(0), where *R*(*H*) is the sheet resistance at a field of *H*, *R*(0) is the sheet resistance at zero field. One can see that MR is positive and may saturate at a large field. Different from ferromagnetic semiconductors or metals, there is no hysteresis in MR curves for the Mn_5_Ge_3_/Ge hybrids. In this case, the spin scattering should have a very small contribution to transport. Similar positive MR effect has also been reported for GeMn-nanocolumns/Ge hybrids [15]. Note that the Ga-doped Ge shows only the neglectable MR effect as shown in Figure 5a. The MR effect can be interpreted by the inhomogeneity of the sample: the different conductivity and Hall resistivity of GeMn and Ge (or Mn-rich and -poor regions). We modeled the hybrid system where Mn-rich nanoparticles were embedded in the Ge matrix by a 2D slice as in the work of Yu et al*.*[31]. Under steady-state conditions, the continuality of the current requires that $\nabla \cdot \left[\sigma \cdot \nabla U\left(x,y\right)\right]=0$, where $U\left(x,y\right)$ is the electrostatic potential at position *(x, y)* on the 2D slice. The materials were fully characterized by their conduction matrices, which vary with the position of different materials. Thus, the finite element method (FEM) proposed by Moussa et al. [32] was used. We applied a constant potential between two electrodes and calculated the induced averaged potential difference at the other two electrodes in the geometry of the van der Pauw method. The current normal to the boundary of the slice was set to zero (the natural boundary condition). The transport properties of the matrix and nanocrystal are simple, characterized by the conductivity matrix with the components:

$$\;\sigma_{\mathrm{xx}}\left(\beta \right)=\sigma_{\mathrm{yy}}\left(\beta \right)=\frac{\sigma \left(0\right)}{\left[1+\beta^{2}\right]}\text{,}$$

$$\;\sigma_{\mathrm{xy}}\left(\beta \right)=-\sigma_{\mathrm{yx}}\left(\beta \right)=-\frac{\sigma \left(0\right)\beta }{\left[1+\beta^{2}\right]}\text{,}$$

where *σ*(0) is the zero-field conductivity and *β* = *R*_*H*_*σ*(0)*μ*_0_*H*, in which *R*_*H*_ is the Hall coefficient of the materials and *μ*_0_ is the susceptibility in vacuum.

The material parameters of the matrix are chosen to be $\;\;\sigma_{H}^{\mathrm{Ge}}\left(0\right)=10^{4}\Omega m^{-1}$, $R_{H}^{\mathrm{Ge}}=10^{-6}\;C^{-1}\;m^{3}$. The material parameters of the nanocrystal are as follows: $\sigma_{H}^{\mathrm{GeMn}}\left(0\right)=a\sigma_{H}^{\mathrm{Ge}}\left(0\right)$ and $R_{H}^{\mathrm{GeMn}}=bR_{H}^{\mathrm{Ge}}$. There are two free parameters *a* and *b* which are the ratios of conductivity and Hall coefficient of the two phases, respectively. Both the conductivity and the Hall coefficient are functions of temperature. The resistance of the system is calculated by FEM where a constant current is applied and the corresponding voltages are measured in the geometry of the van der Pauw method. The calculated curves are presented in Figure 5b. The experimental MR curves can be well reproduced by FEM calculations. The *a* and *b* values used in the FEM calculations are shown in Figure 5c. The MR magnitude is sensitive to the ratio of conductivity of the two constitutes. Beside the magnetoresistance, the samples also show anomalous Hall resistance (i.e., the Hall resistance deviates from a linear behavior), which can be explained by two kinds of carriers with different mobilities [33]. On the other hand, we have to note the rather large discrepancy in the MR magnitude between the experimental and modeled values. In the model, for simplifying, we neglect the anomalous Hall effect in the GeMn constitute, which may induce this discrepancy. Also, in order to account non-monotonic dependence of MR on temperature (see Figure 5c), we have to vary parameters *a* and *b* accordingly. The decrease of *a* and *b* at temperature below 50 K cannot be understood and is the aim for the future work.

## Conclusions

We have prepared magnetic Mn_5_Ge_3_ nanocrystals embedded inside the Ge matrix by Mn ion implantation into Ge substrates. The crystalline size of Mn_5_Ge_3_ can be tuned by varying the Mn fluence. The Mn ions in Mn_5_Ge_3_ nanocrystals are in the 3*d*^6^ configuration. Large positive magnetoresistance has been observed in the Mn_5_Ge_3_/Ge hybrid system. It could be due to the inhomogeneity in samples with constitutes having different transport properties.

## Competing interests

The authors declare that they have no competing interests.

## Authors’ contributions

SZ designed the experiments and wrote the manuscript. WXZ and ZH made fittings for the magnetoresistance data. AS performed the XRD measurement. YW carried out the XMCD and XAS measurements. DB and HS helped during magneto-transport measurement. AM performed the TEM characterization. WLZ supervised the fitting of the magnetoresistance data. MH supervised the whole work. All authors read and approved the final manuscript.
